# Insights From the Genome Sequence of *Mycobacterium paragordonae*, a Potential Novel Live Vaccine for Preventing Mycobacterial Infections: The Putative Role of Type VII Secretion Systems for an Intracellular Lifestyle Within Free-Living Environmental Predators

**DOI:** 10.3389/fmicb.2019.01524

**Published:** 2019-07-03

**Authors:** Byoung-Jun Kim, Ga-Yeong Cha, Bo-Ram Kim, Yoon-Hoh Kook, Bum-Joon Kim

**Affiliations:** Department of Microbiology and Immunology, Biomedical Sciences, Liver Research Institute, Institute of Endemic Diseases, Medical Research Center, Seoul National University College of Medicine, Seoul, South Korea

**Keywords:** *Mycobacterium paragordonae*, genome sequence, lateral gene transfer, Type VII secretion systems, *M. gordonae*

## Abstract

*Mycobacterium paragordonae* (Mpg) is a temperature-sensitive *Mycobacterium* species that can grow at permissive temperatures but fails to grow above 37°C. Due to this unique growth trait, Mpg has recently been proposed as a novel live vaccine candidate for the prevention of mycobacterial infections. Furthermore, the increasing frequency of the isolation of Mpg from water supply systems led us to hypothesize that the free-living amoeba system is the natural reservoir of Mpg. In this study, we report the complete 6.7-Mb genome sequence of Mpg and show that this genome comprises four different plasmids with lengths of 305 kb (pMpg-1), 144 kb (pMpg-2), 26 kb (pMpg-3), and 17 kb (pMpg-4). The first two plasmids, pMpg-1 and -2, encode distinct Type VII secretion systems (T7SS), ESX-P5 and ESX-2, respectively. Genome-based phylogeny indicated that Mpg is the closest relative to *M. gordonae*, which has a 7.7-Mb genome; phylogenetic analysis revealed an average of 86.68% nucleotide identity between these two species. The most important feature of Mpg genome is the acquisition of massive genes related to T7SS, which may have had effect on adaptation to their intracellular lifestyle within free-living environmental predators, such as amoeba. Comparisons of the resistance to bacterial killing within amoeba indicated that Mpg exhibited stronger resistance to amoeba killing compared to *M. gordonae* and *M. marinum*, further supporting our genome-based findings indicating the special adaptation of Mpg to free-living amoeba. We also determined that, among the strains studied, there were more shared CDS between *M. tuberculosis* and Mpg. In addition, the presence of diverse T7SSs in the Mpg genome, including an intact ESX-1, may suggest the feasibility of Mpg as a novel tuberculosis vaccine. Our data highlight a significant role of lateral gene transfer in the evolution of mycobacteria for niche diversification and for increasing the intracellular survival capacity.

## Introduction

*Mycobacterium paragordonae* (Mpg) is a slow growing, scotochromogenic non-tuberculous mycobacteria (NTM) that prefers a lower temperature for growth (28°C to 30°C) and is phylogenetically closest to *M. gordonae* (Kim B.J. et al., 2014). Mpg exhibits distinct temperature-sensitive growth and fails to grow above 37°C. Higher temperatures lead to the failure of Mpg to replicate, e.g., at deeper regions within the body in *in vivo* challenges, guaranteeing its feasibility as a safe live bacterial vaccine vehicle. Indeed, we previously demonstrated that a live Mpg strain exerted enhanced protective vaccine efficacies against mycobacterial infections such as *Mycobacterium tuberculosis* or *M. abscessus* in vaccinated mice, compared to BCG (Kim et al., 2017).

Free-living amoeba (FLA) have been frequently isolated from habitats common to mycobacteria (Thomas and McDonnell, 2007; Falkinham, 2009), including cold drinking water distribution systems (Eddyani et al., 2008; Thomas et al., 2008), hot water systems in hospitals (von Reyn et al., 2002), and cooling towers (Pagnier et al., 2008). Several lines of evidence indicate the infection of *Acanthamoeba* FLA with both pathogenic and environmental mycobacteria, such as *M. avium* subsp. *paratuberculosis*, *M. intracellulare*, and *M. bovis* (Taylor et al., 2003; Adekambi et al., 2006; Samba-Louaka et al., 2018). In addition, isolated FLAs, have also been reported to be associated with various mycobacterial species, including *M. gordonae*, *M. xenopi*, *M. avium*, and *M. kansasii*, in hospital water (Cirillo et al., 1997; Steinert et al., 1998; Vaerewijck et al., 2005; Thomas et al., 2008). These findings strongly support the notion of an “endosymbiotic” relationship between mycobacteria and the host FLA (Drancourt et al., 2007; Iovieno et al., 2010; Glaser et al., 2011). In this model, the host protozoa would theoretically protect phagocytized mycobacteria from adverse environmental insults, including extreme temperature, drought and diverse biocide attacks via cyst formation (Barker and Brown, 1994; Ben Salah and Drancourt, 2010; Denoncourt et al., 2014). Moreover, mycobacteria could also make use of the protozoan nutrients (Thomas and McDonnell, 2007). Overall, FLA could contribute to the survival of intracellular mycobacteria by providing an environmental niche for persistent infection and by acting as a transmission vector.

In the proposed mycobacterial evolutionary scenario in water and in soil, the most recent common ancestor (MRCA) of mycobacteria may have encountered free-living predators such as FLA (Salah et al., 2009). Mycobacteria have evolved various strategies to resist such unicellular predators, including the capacity to avoid phagocytosis and to replicate intracellularly within the protozoa (Medie et al., 2011). Over time, as mycobacteria diverged from the MRCA, they have been further evolved to resist destruction by the infected host macrophage or dendritic cell (DC), which is a key step for pathogenicity (Salah et al., 2009). Thus, a better understanding of the evolution and biology of mycobacteria and their adaptation to the intracellular lifestyle within FLA via a genome-based approach is necessary to elucidate the virulence mechanisms of pathogenic mycobacteria and to develop treatment strategies.

Although the exact natural habitat of Mpg remains a mystery, after our initial description of Mpg (Kim B.J. et al., 2014), the isolation of this species from water supply systems has recently increased worldwide (Azadi et al., 2016, 2017). Therefore, together with the distinct preference of Mpg for lower temperatures, the recent epidemiologic study suggests that Mpg may make use of FLA as a natural reservoir or a transmission vector.

Here, we have introduced the complete genome sequences of Mpg, the type strain *M. paragordonae* JCM 18565^T^. This genome-wide comparison of Mpg with the genomes of *M. gordonae* and *M. marinum* provides deeper insight into the biology of Mpg as an environmental generalist and discloses the evolutionary history among these three closely related but clearly distinct species.

## Results and Discussion

### Genome Sequencing and General Features of the Mpg Genome

**FIGURE 1:**
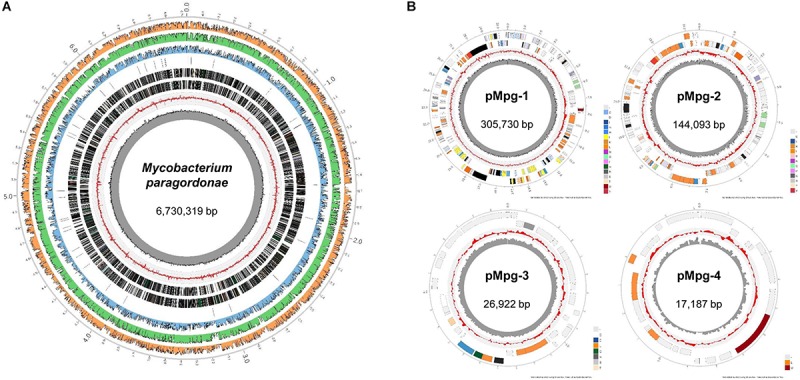
Circular representation of the Mpg genome and plasmids. Whole-genome sequencing of Mpg revealed that Mpg harbors **(A)** a single circular chromosome (6,730,319 bp) and **(B)** four plasmids (pMpg-1, 305,730 bp; pMpg-2, 144,093 bp; pMpg-3, 26,922 bp; pMpg-4, 17,187 bp). From inside to outside, track 1 (black peaked line) indicates the GC content, and track 2 (red peaked line) indicates the GC skewness of the Mpg genome and plasmids. Track 3 and 4 represent the predicted ORFs in the reverse and forward orientation, respectively. In the case of the Mpg genome, track 5 indicates the locations of tRNAs. Tracks 6 through 8 represent the sequence identities compared with *M. tuberculosis* H37Rv^T^, *M. gordonae* DSM 44160^T^, and *M. marinum* M, respectively.

The Mpg genome sequence was obtained using a Pacific Biosciences RS sequencer and an Illumina Hi-Seq sequencer. A total of approximately 87,929,586 reads were obtained, comprising more than ∼1,288.2× coverage of the estimated 6.7 Mb of the Mpg genome. The sequencing data revealed that Mpg has a circular chromosome of 6,730,319 bp (GenBank accession no. CP025546) and four circular plasmids with lengths of 305,730 bp (pMpg-1, CP025547), 144,093 bp (pMpg-2, CP025548), 26,922 bp (pMpg-3, CP025549), and 17,187 bp (pMpg-4, CP025550) (Figure 1 and Tables 1, 2). The genome contains 6,265 predicted ORFs, a single rRNA operon and 47 tRNAs; the four plasmids (pMpg-1 thorough 4) contain 284, 145, 30, and 20 ORFs, respectively. Among Mpg, *M. gordonae* and two pathogenic mycobacterial strains, *M. tuberculosis* and *M. marinum*, Mpg exhibited a higher number of ORFs (6,265 ORFs) compared with the two pathogenic *M. tuberculosis* (4,086 ORFs) and *M. marinum* (5,604 ORFs) strains. However, *M. gordonae*, which is genetically related to Mpg, contains more ORFs (6,915 ORFs) compared with Mpg. The G+C content (67.03%) of the Mpg genome was shown to be higher than that of the pathogenic mycobacterial strains *M. tuberculosis* (65.6%) and *M. marinum* (65.7%); however, *M. gordonae* has a percentage of G+C content (66.8%) similar to that of Mpg. The identified plasmids exhibited a lower percentage of G+C content (64.7 to 65.7%) compared with that of the genome from Mpg, except for the pMpg-4 plasmid (67.9%) (Figure 1 and Tables 1, 2). Overall, our genome data indicate that Mpg may have evolved from a more generalist species, *M. gordonae*, via chromosome genome reduction and the acquisition of diverse plasmids by lateral gene transfer (LGT).

**TABLE 1:** General genomic features of Mpg compared with three other mycobacteria.

	***M. paragordonae* JCM 18565^T^**	***M. gordonae* DSM 44160^T^**	***M. marinum* M**	***M. tuberculosis* H37Rv**
Chromosome size (base pairs)	6,730,319	7,601,632	6,660,144	4,411,532
G+C (%)	67.0	66.8	65.7	65.6
Protein-coding sequences (CDS)	6,265	6,915	5,604	4,086
Average CDS length	1,013	984.5	1,073	977
rRNA	1	1	1	1
tRNA	47	48	46	45

**TABLE 2:** General plasmid features of Mpg.

	**pMpg-1**	**pMpg-2**	**pMpg-3**	**pMpg-4**
Chromosome size (base pairs)	305,730	144,093	26,922	17,187
G+C (%)	65.7	65.3	64.7	67.9
Protein-coding sequences (CDS)	284	145	30	20
Average CDS length	946	915	678	697

### Phylogenetic Relationships Based on the Mpg Genome

**FIGURE 2:**
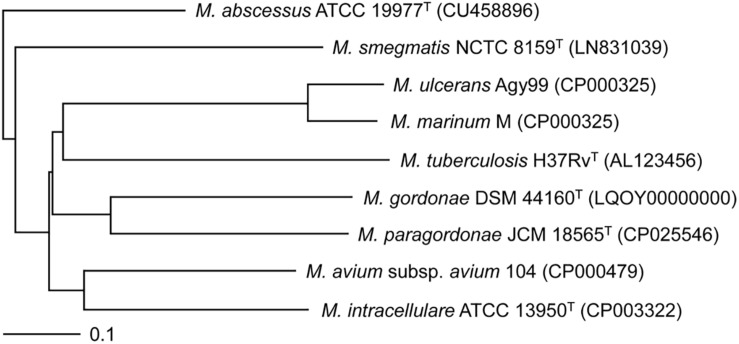
Phylogenetic tree based on the whole-genome sequences of Mpg and other mycobacterial strains. The tree was constructed using the neighbor-joining method using the MAUVE Genome Alignment software and visualized using the TreeViewX program. The bar indicates the number of substitutions per nucleotide position.

Using the genome sequence of Mpg, a phylogenetic analysis was conducted which included other mycobacterial genome sequences. A genome-based phylogenetic tree showed that Mpg was grouped together with *M. gordonae* DSM 44160^T^, which was previously demonstrated to be genetically close to Mpg (Kim B.J. et al., 2014). Moreover, Mpg was clustered with pathogenic mycobacterial strains, such as *M. marinum* M, *M. ulcerans* Agy99, and *M. tuberculosis* H37Rv^T^ (Figure 2). The average nucleotide identity (ANI) value between Mpg and *M. gordonae* DSM 44160^T^ was 88.68%, which was below the recommended cut-off value of 95 to 96% ANI for species delineation (Goris et al., 2007; Richter and Rossello-Mora, 2009; Kim M. et al., 2014). However, the ANI value between Mpg and *M. gordonae* was higher than that between Mpg and *M. marinum* M (78.12%) and between Mpg and *M. tuberculosis* H37Rv (78.58%) (Supplementary Table S1). Therefore, these findings are in agreement with our previous report showing that Mpg is a distinct species within the genus *Mycobacterium* and is phylogenetically closest to *M. gordonae* (Kim B.J. et al., 2014).

### Functional Classification of Mpg Proteins

To functionally classify the predicted ORFs of the Mpg genome, the amino acid sequences of Mpg proteins were analyzed using the BLAST program against the COG database, which serves as a platform for the functional annotation of prokaryotic genomes (Tatusov et al., 2003). Based upon the similarity with the COG database, 65.9% of the Mpg proteins were assigned COG functions; however, 34.1% of the Mpg proteins were not annotated in the COG database. The COG analysis of the Mpg genome revealed that 31.96% of the Mpg proteins belong to the “Metabolism” category, 10.43% belong to “Information storage and processing” (ISP), 10.45% belong to “Cellular processes and signaling” (CPS), and 13.06% of the proteins were ‘Poorly categorized’ proteins (Supplementary Figure S1).

A COG-based comparative analysis of the gene distribution among the genomes of Mpg, *M. gordonae* DSM 44160^T^, *M. marinum* M, and *M. tuberculosis* H37Rv^T^ revealed that these strains have similar proportions of COG-based functional classifications. However, there were certain variations in each functional category. In the ‘ISP’ category, the pathogenic mycobacterial strains *M. marinum* M and *M. tuberculosis* H37Rv^T^ exhibited a higher proportion of genes involved in ‘Translation, ribosomal structure and biogenesis’ (J, 27.52 and 27.08%, respectively) compared to the Mpg and *M. gordonae* DSM 44160^T^ strains (22.14 and 21.29%, respectively). Additionally, the Mpg and *M. gordonae* DSM 44160^T^ strains (50.08 and 50.29%, respectively) exhibited a higher proportion of genes related to ‘Transcription’ (K) compared with the *M. marinum* M and *M. tuberculosis* H37Rv^T^ strains (48.74 and 35.04%, respectively) (Supplementary Figure S2). Overall, the functional classification of the genome indicated that the general function of the Mpg genome is similar to that of the generalist *M. gordonae* but distinct from the more specialist group, *M. marinum* and *M. tuberculosis.*

### Comparative Genomic Analysis Among the Genome Sequences of Mpg, *M. gordonae*, *M. marinum*, and *M. tuberculosis*

Our synteny analysis of the Mpg genome structure (6.7 Mb) compared with that of *M. marinum* M (6.6 Mb) and *M. tuberculosis* H37Rv (4.4 Mb) revealed a relatively high conservation of genome size and gene order between the genomes of Mpg and *M. marinum* M; however, between the genomes of Mpg and *M. tuberculosis* H37Rv, there were two large loci which exhibited reversed gene orientation, and genome reduction was also detected (Supplementary Figure S3).

**FIGURE 3:**
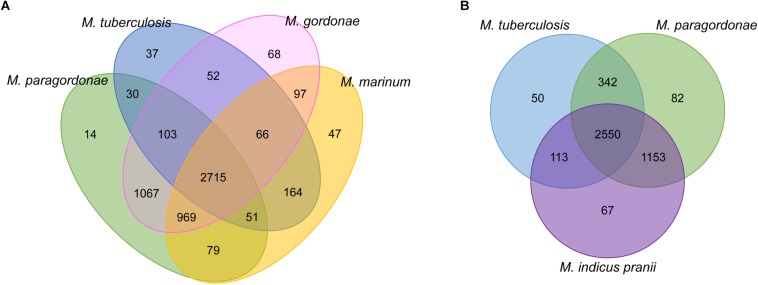
Venn diagrams showing orthologous CDS among Mpg and other mycobacterial species as determined by BLASTCLUST analysis (0.8 of length coverage threshold). **(A)** Comparison among the Mpg, *M. gordonae* DSM 44160^T^, *M. marinum* M, and *M. tuberculosis* H37Rv^T^ strains. **(B)** Comparison among the Mpg, *M. tuberculosis* H37Rv^T^, and *M. indicus pranii* strains.

To assess the number of genes that are shared between each genome, a web-based program, OrthoVenn was used to analyze the protein sequences of Mpg, *M. gordonae*, *M. marinum*, and *M. tuberculosis*. The results are summarized in a Venn diagram, which shows the CDS that were both conserved and unique between these species (Figure 3A). Mpg shares more CDS (4,854/5,028 CDS, 96.5%) with *M. gordonae* DSM 44160^T^ compared with *M. marinum* M (3,814/5,028 CDS, 75.9%) or *M. tuberculosis* H37Rv (2,899/5,028 CDS, 57.7%). Based on the numbers of shared CDS, Mpg is more closely related to *M. marinum* M than to *M. tuberculosis* H37Rv. This finding supports our genome synteny analysis among the genomes of Mpg, *M. marinum* M, and *M. tuberculosis* H37Rv (Supplementary Figure S1). However, the ANI value between the genomes of Mpg and *M. tuberculosis* (78.58%) was slightly higher than that between Mpg and *M. marinum* M (78.12%) (Supplementary Table S1).

*Mycobacterium indicus pranii* (MIP), which was formerly known as *Mycobacterium w*, is non-pathogenic and is classified in Runyon group IV (Reddi et al., 1994). MIP has been evaluated as a candidate for leprosy and tuberculosis vaccines (Gupta et al., 2009) due to the presence of several B and T cell determinants common with *M. leprae* (Yadava et al., 1991). As indicated in Figure 3B, Mpg shares more CDS (342 ORFs) with *M. tuberculosis* than with MIP (113 ORFs). Additionally, the average sequence similarity of shared CDS between Mpg and *M. tuberculosis* was 88%, which was slightly higher than that of the shared CDS between MIP and *M. tuberculosis* (87%). These results suggest that Mpg may have advantages over MIP for use as a live tuberculosis vaccine.

### ESX Locus of the Mpg Chromosome

**FIGURE 4:**
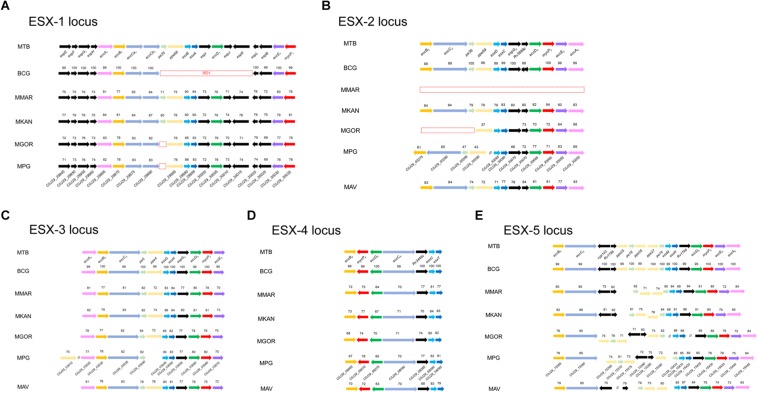
Comparison of the genetic organization of the ESX loci in various mycobacterial genomes, including Mpg. Comparison of the genetic organization of the ESX loci identified in the genomes of *M. tuberculosis*, BCG, *M. marinum*, *M. kansasii*, *M. gordonae*, *M. avium*, and Mpg. **(A)** ESX-1 locus. **(B)** ESX-2 locus. **(C)** ESX-3 locus. **(D)** ESX-4 locus. **(E)** ESX-5 locus. The sequence similarities with *M. tuberculosis* are indicated for each ORF. The red dashed box indicates the deleted genes. The red lined box in panel **(B)** indicates the separation of the genes involved in the ESX-2 locus, and their location is also indicated above the box.

The type VII secretion system (T7SS) has been reported to play a pivotal role in the intracellular survival and host infection of mycobacteria, including *M. tuberculosis*. All five ESX loci (ESX-1, ESX-2, ESX-3, ESX-4, and ESX-5) are intact in the genome of *M. tuberculosis* (Cole et al., 1998; Tekaia et al., 1999; Gey van Pittius et al., 2001), but attenuated BCG strains lack RD1 of the ESX-1 locus (Hsu et al., 2003; Lewis et al., 2003). Interestingly, although Mpg has relatively low sequence similarities compared to *M. tuberculosis* orthologs, Mpg contains all five types of ESX loci in its genome (Figure 4). In the ESX-1 locus of Mpg, except for an ortholog of PE35, all of the components (19 ORFs, C0J29_29945∼30035) are conserved with 69 to 85% sequence similarity compared to *M. tuberculosis*. The effector molecules ESAT-6 (*esxA*) and CFP-10 (*esxB*), which are lost in BCG, are also intact in the Mpg genome, showing relatively high sequence similarities with those of *M. tuberculosis* (83 and 86%, respectively), suggesting an advantage of Mpg over BCG as a tuberculosis vaccine (Figure 4A). The ESX-1 system is also found in a range of mycobacteria, including pathogenic mycobacteria, such as *M. kansasii* (Sorensen et al., 1995), *M. leprae* (Cole et al., 2001) *M. marinum* (Stinear et al., 2008), and the saprophytic *M. smegmatis* (Coros et al., 2008). However, the live tuberculosis vaccine, *M. bovis* BCG (BCG) strain lacks *esxA*, owing to spontaneous deletions of the ESX-1 locus, which is known as region of difference 1 (RD1) (Mahairas et al., 1996). Given that the ESX-1 system is essential for resistance to and evasion of the host immune system and also contributes to the high antigenicity of *M. tuberculosis*, the presence of a nearly intact ESX-1 system in the genome of Mpg might be likely to contribute to survival within the host. However, PE35 was not found in the ESX-1 locus of Mpg genome. This gene has recently been shown to play an important role in secretion of the ESX-1 substrate, EsxA (Abdallah et al., 2019). So, to explain the direct relationship between the ESX-1 locus of Mpg genome and their intracellular survival, additional analyses using ESX-1 deletion mutant should be conducted in the future study. Actually, in the previous report (Pym et al., 2003), recombinant BCG subject to reintroduction of the RD-1 complete locus resulted in specific ESAT-6-dependent immune responses and demonstrated better protection against challenge with *M. tuberculosis* in vaccinated mice, showing less severe pathology and reduced dissemination of the pathogen, as compared with control animals immunized with BCG alone. It highlights the importance of ESX-1 gene in tuberculosis vaccine and also suggests the potential use of Mpg as a novel tuberculosis vaccine.

Of the five ESX loci (Figure 4), a pronounced difference between Mpg, *M. gordonae*, and *M. marinum* was found in the ESX-2 locus. The ESX-2 locus was also shown to be intact in the Mpg genome. However, none of the orthologs of ESX-2 were found within the *M. marinum* M genome, and only the partial orthologs of the ESX-2 were detected in the *M. gordonae* genome. Notably, the ESX-2 genes of Mpg were separated into two distantly located regions in its genome, unlike the case of *M. tuberculosis*, in which all ESX-2 genes (corresponding ORFs are Rv3884c∼Rv3895c) are located in one region proximal to the ESX-1 locus (corresponding ORFs are Rv3869∼Rv3883c). In the case of Mpg, the first part of ESX-2 (8 ORFs corresponding to C0J29_30050∼30085) is located next to the ESX-1 locus, but the second part of ESX-2 (corresponding to C0J29_00275∼00290) is located distantly from the ESX-1 locus (Figure 4B). Given that the genes corresponding to *pe36*, *eccC2*, and *eccB2* in the second region of Mpg were not detected in the *M. gordonae* genome, and the sequence similarity values were very low compared to those of the first part of Mpg, the second part of the ESX-2 genes of Mpg appears to be acquired by lateral gene transfer from other bacteria after the gene loss of *M. gordonae*.

### The Presence of Genes Encoding ESX Systems on Two Plasmids of Mpg

**FIGURE 5:**
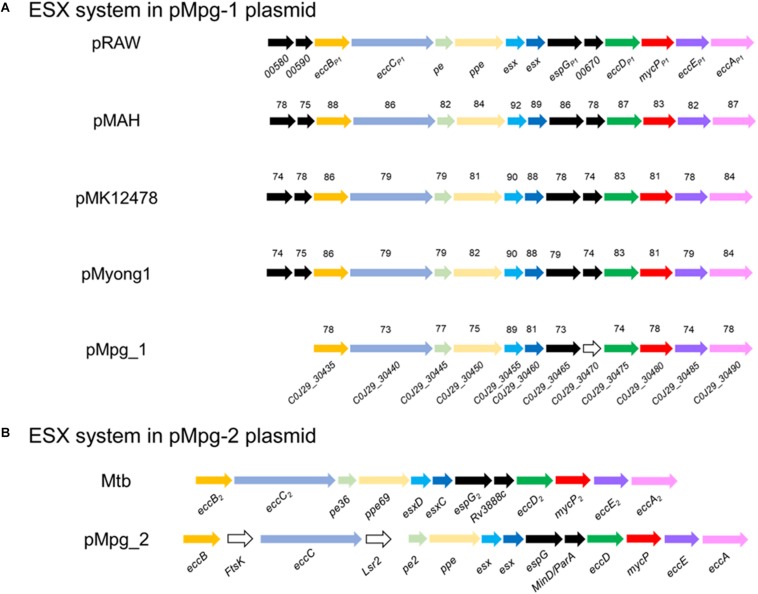
Comparison of the genetic organization of the ESX loci in various mycobacterial plasmids, including Mpg. **(A)** The ESX-P5 locus from pMpg-1 was compared with other loci in the mycobacterial plasmids pRAW (*M. marinum* E11), pMAH135 (*M. avium* subsp. *hominissuis* TH135), pMK12478 (*M. kansasii* ATCC 12478), and pMyong1 (*M. yongonense*). **(B)** The ESX-2-like locus in pMpg-2 was compared with the ESX-2 locus of *M. tuberculosis*. In panel **(A)**, the sequence similarities are indicated for each ORF, which were calculated against genes from pRAW. Genes which were not matched with those of pRAW and additional insertions were represented as white arrows.

In addition to the known chromosomal ESX loci of mycobacterial strains, the existence of plasmid-encoded ESX systems was recently reported (Ummels et al., 2014). Apart from the five types of intact ESX loci in the Mpg chromosome, we found two additional distinct ESX on the pMpg-1 and -2 plasmids, respectively. On the pMpg-1 plasmid, the putative ESX locus (12 ORFs corresponding to C0J29_30435∼30490) exhibited a gene orientation similar to that of recently identified mycobacteria plasmid-encoded ESX-P5 systems (Ummels et al., 2014; Newton-Foot et al., 2016; Figure 5A). On the pMpg-2 plasmid, another putative ESX system was also identified; however, this system has no homology with any ESX system derived from mycobacterial plasmids. Instead, it has an orientation similar to the chromosomal ESX-2 system in the genome of *M. tuberculosis* (Figure 5B and Supplementary Table S2). These results suggest that the ESX system on the pMpg-2 plasmid may be an ancient ESX system that evolved into ESX-P2.

To examine the phylogenetic relationship of the two ESX loci from the pMpg-1 and -2 plasmids, various EccC protein sequences from mycobacterial chromosomes (from ESX-1 to ESX-5 of *M. marinum* and *M. tuberculosis*) and plasmids (ESX-P1 through -P3 and -P5) were aligned, and an alignment-based phylogenetic tree was constructed. As indicated above, the EccC protein from the pMpg-1 plasmid was grouped with those of mycobacterial ESX-P5 [*M. yongonense* (pMyong1), *M. kansasii* (pMK12478), *M. marinum* (pRAW), and *M. avium* subsp. *hominissuis* (pMAH135)]. The EccC protein from the pMpg-2 plasmid was clustered with the EccC protein of the chromosomal ESX-2 locus in *M. tuberculosis*, not with the plasmid-derived ESX-P2 system (Supplementary Figure S4).

### Enhanced Resistance of Mpg to Bacterial Killing by Amoeba

**FIGURE 6:**
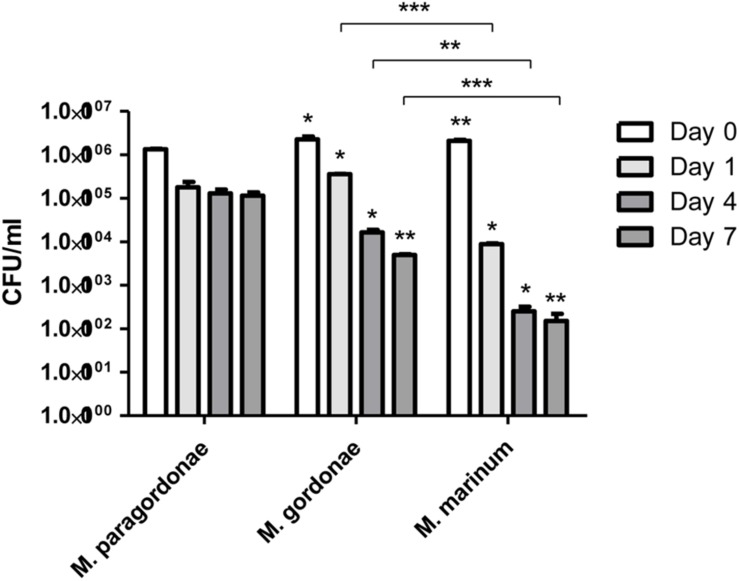
CFU enumeration of Mpg, *M. gordonae* and *M. marinum* from infected *A. castellanii*. CFU counts of Mpg, *M. gordonae* and *M. marinum* after infection of *A. castellanii* at a M.O.I. of 10 for 7 days. Statistical analyses (Student *t*-test) were performed among the CFUs of Mpg, *M. gordonae* and *M. marinum* at each infection time-point (^*^P < 0.05 and ^∗∗^ *P* < 0.01). Asterisks above each bar of *M. gordonae* and *M. marinum* indicate the statistical significance between *M. paragordonae* and those two strains at each time point.

Our genome data on diverse T7SS systems, along with the high isolation frequency of Mpg from water supply systems worldwide and the unique growth traits of Mpg (its failure to grow above 37°C), led us to hypothesize that Mpg could make use of FLA systems as its natural reservoir and could resist phagocytic death. To address this issue, we compared the intracellular survival capacity against the amoeba (*Acanthamoeba castellanii*) phagocytic killing mechanism between Mpg, *M. gordonae* and *M. marinum* via coculturing with *A. castellanii* for 7 days. Before the test for survival ability in amoeba, the growth rate of three mycobacterial strains, Mpg, *M. gordonae* and *M. marinum* was determined in 7H9 broth media for 7 days. These strains showed similar growth rate until 5 days, however, *M. marinum* showed slightly slow growth rate at 30°C (Supplementary Figure S5). At the indicated time-points (0, 1, 4, and 7 days) of the infection experiment, the viability of Mpg, *M. gordonae* and *M. marinum* was compared by counting the colony forming units (CFUs) of each strain. Viable Mpg, *M. gordonae* and *M. marinum* were detected at all time-points. Mpg showed lower CFU [(133.50 ± 4.95) × 10^4^ CFUs] at the infection time of Day 0, than *M. gordonae* [(226.00 ± 36.77) × 10^4^ CFUs] and *M. marinum* [(209.00 ± 11.31) × 10^4^ CFUs]. Also, bacterial viability of the three strains was decreased in a time-dependent manner. However, CFUs [(1155.00 ± 219.20) × 10^2^ CFUs] of Mpg was maintained 7 days after infection compared to those of *M. gordonae* [(45.50 ± 2.12) × 10^4^ CFUs] and *M. marinum* [(1.5 ± 0.71) × 10^2^ CFUs]. This finding indicates that Mpg may have an advantage in resistance to phagocytic death after its infection of amoeba (Figure 6).

## Conclusion

We conducted a comparative genome analysis of Mpg with evolutionarily close species, such as *M. gordonae*, *M. marinum*, or *M. tuberculosis* to gain insight into questions regarding the potential use of Mpg as a novel tuberculosis vaccine candidate and its frequent isolation from water supply systems, such as tap waters; we primarily focused on T7SS systems. Our data indicate that Mpg genome has acquired massive genes related to T7SS, and these genes may have contributed to the adaptation of Mpg to intracellular lifestyle within free-living environmental predators, such as amoeba. Comparison of the survival capacity within amoeba indicated that Mpg exhibited the better resistance to bacterial killing within amoeba, compared with *M. gordonae* or *M. marinum*, further supporting our genome-based finding of the special adaptation of Mpg to free living amoeba. Taken together, our data highlight a significant role for lateral gene transfers in mycobacteria evolution for niche diversification and for adapting to intracellular survival.

## Materials and Methods

### Genome Sequencing

The genomic DNA of Mpg was sequenced using a Pacific Biosciences RS sequencer (300,584 and 5,647 reads) and an Illumina Hi-Seq sequencer (105,177,988 reads). PacBio raw data was assembled *de novo* with the hierarchical genome assembly process (HGAP) of the single molecule real-time (SMRT) analysis software (Pacific Biosciences, United States). After the *de novo* assembly, a total of 6 contigs were obtained, which were corrected by mapping using the CLC reference assembler compared with the Illumina Hi-Seq raw data. There were 87,929,586 total mapped reads, representing ∼1,288.2 × coverage for the estimated 6.7-Mb genome. All of the remaining gaps between contigs were filled using gap-filling PCR amplification. After obtaining the genome sequence, gene prediction was performed using the NCBI Prokaryotic Genome Annotation Pipeline (PGAP)^1^ (Tatusova et al., 2016; Haft et al., 2018). The sequencing analysis was performed in the National Instrumentation Center for Environmental Management (NICEM) (Genome Analysis Unit) at Seoul National University. The GenBank accession numbers of the sequences are CP025546 to CP025550.

### Genome Sequence-Based Phylogenetic Analysis

Using the genome sequences of Mpg (GenBank accession No. CP025546∼CP025550), *M. tuberculosis* H37Rv (AL123456), *M. gordonae* DSM 44160^T^ (LQOY00000000), *M. marinum* M (CP000325), *M. ulcerans* Agy99 (CP000325), *M. avium* subsp. *avium* 104 (CP000479), *M. intracellulare* ATCC 13950^T^ (CP003322), *M. smegmatis* NCTC 8159^T^ (LN831039), and *M. abscessus* ATCC 19977^T^ (CU458896), genome sequence based phylogenetic analysis was conducted. These genome sequences were subjected to whole-genome multiple sequence alignments using the neighbor-joining method with the Mauve Genome Alignments software^2^ (Darling et al., 2004). A phylogenetic tree was generated using the aligned genome sequences and visualized with the TreeView X program^3^ (Yoon et al., 2017). Additionally, using the genomes of Mpg, *M. gordonae*, *M. marinum*, and *M. tuberculosis* and Mpg, *M. tuberculosis*, and *M. indicus pranii*, Venn diagrams were constructed using the web-based program, OrthoVenn (Wang et al., 2015). An *E*-value cut off of 1e^–5^ was used for protein similarity comparisons. An inflation value of 1.5 was used for the generation of orthologous clusters. Also, a neighbor-joining phylogenetic tree based on the EccC protein sequences of the pMpg-1 and -2 plasmids and the various EccC proteins from mycobacterial chromosomes (ESX-1 through -5 of *M. marinum* and *M. tuberculosis*) and plasmids (ESX-P1, pMFLV01 of *M. gilvum* PYR-GCK; ESX-P2, pMKMS01 of *Mycobacterium* sp. KMS and plasmid 2 of *M. abscessus* subsp. *massiliense* 50594; ESX-P3, pMKMS02 of *Mycobacterium* sp. KMS and plasmid 1 of *Mycobacterium* sp. MCS; ESX-P5, pMyong1 of *M. yongonense*, pMK12478 of *M. kansasii*, pRAW of *M. marinum* and pMAH135 of *M. avium* subsp. *hominissuis*) was constructed with the MEGA7 software.

### Strains Used in This Study

The *M. paragordonae* JCM 18565^T^ (Kim B.J. et al., 2014), *M. gordonae* ATCC 14470^T^, and *M. marinum* JCM 17638^T^ strains were used in this study. All strains were cultured from low-passaged frozen stocks (at -70°C) to the exponential phase and subcultured in Middlebrook 7H9 broth supplemented with albumin dextrose catalase (ADC) and on Middlebrook 7H10 agar plates supplemented with oleic albumin dextrose catalase (OADC) for 2 weeks at 30°C and/or 37°C. To obtain single bacterial cell suspensions, all strains were suspended in PBS with 0.05 % Tween 80 (PBS-T) and passed through a 27-gauge needle three to five times. The growth rate of Mpg, *M. gordonae* and *M. marinum* was determined by optical density (OD) at 600 nm after culture in 7H9 broth supplemented with ADC for 7 days.

### *Acanthamoeba castellanii* Infection and CFU Assays

To evaluate the survival of Mpg, *M. gordonae* and *M. marinum* within the infected amoeba, the *A. castellanii* strain ATCC 30234 was cultured and maintained in PYG medium [20 g/L of proteosepeptone, 1 g/L of yeast extract, 1 g/L of sodium citrate, 0.1 M glucose, 0.4 mM CaCl_2_, 4 mM MgSO_4_, 2.5 mM Na_2_HPO_4_, 2.5 mM KH_2_PO_4_, 50 μM Fe(NH_4_)_2_(SO_4_)_2_, pH 6.5] at 28°C. The cultured *A. castellanii* cells were seeded in a 6-well plate at 7.5 × 10^5^ cells per well, and then the cells were infected with the Mpg, *M. gordonae* and *M. marinum* strains at an M.O.I. of 10 for 4 h at 28°C. Infected amoeba were washed and resuspended in 1 mL of fresh PAS buffer (Page’s amoeba saline; 1 g/L of sodium citrate, 0.4 mM CaCl_2_, 4 mM MgSO_4_, 2.5 mM Na_2_HPO_4_, and 2.5 mM KH_2_PO_4_) and cultured for 0, 1, 4 and 7 days. At each time-point, the infected amoeba was centrifuged at 800 × *g* for 5 min, after which the supernatant was discarded, the pellet was lysed with 0.5% Triton X-100 (in PBS) and, finally, the homogenized suspensions were diluted and plated onto 7H10 agar plates. The plates were incubated at 28°C for 2 or 3 weeks before the CFUs were counted.

### Statistical Analysis

All presented data are expressed as the mean ± standard deviation (SD). Student’s *t*-test was used to compare the variance using Microsoft Excel software, and the differences were considered statistically significant at probability values less than 0.05. Two independent experiments were conducted and the representative data are presented.

## Author Contributions

ByJ-K and B-RK carried out the genome sequence analyses. G-YC carried out the infection test and generated the recombinant mycobacterial strains. BuJ-K and Y-HK designed and interpreted the results of experiments and sequence analyses. BuJ-K wrote the manuscript.

## Conflict of Interest Statement

The authors declare that the research was conducted in the absence of any commercial or financial relationships that could be construed as a potential conflict of interest.

## Footnotes

1 https://www.ncbi.nlm.nih.gov/genome/annotation_prok/

2 http://darlinglab.org/mauve/mauve.html

3 https://treeview-x.en.softonic.com/
