# Supplementary material for: Insights From the Genome Sequence of Mycobacterium paragordonae, a Potential Novel Live Vaccine for Preventing Mycobacterial Infections: The Putative Role of Type VII Secretion Systems for an Intracellular Lifestyle Within Free-Living Environmental Predators
Source: Front Microbiol. 2019 Jul 3;10:1524. doi: 10.3389/fmicb.2019.01524 (PMC6616192; doi:10.3389/fmicb.2019.01524)
Supplement: Supplementary file 1 [file Data_Sheet_1.PDF]

Supplementary Figures and Tables

Insights from the genome sequence of *Mycobacterium paragordonae*, a novel live vaccine for preventing mycobacterial infections: the putative role of Type VII secretion systems for an intracellular lifestyle within free-living environmental predators

Byoung-Jun Kim[1], Ga-Yeong Cha[1], Bo-ram Kim[1], Yoon-Hoh Kook[1] and Bum-Joon Kim[1] *

Department of Microbiology and Immunology, Biomedical Sciences, Liver Research Institute, Institute of Endemic Diseases, Seoul National University Medical Research Center (SNUMRC), Seoul National University College of Medicine, Seoul 110-799, Republic of Korea[1]

***Author for correspondence:** Bum-Joon Kim, phD, Professor

Department of Microbiology and Immunology, Liver Research Institute and Cancer Research Institute, College of Medicine, Seoul National University. 28 Yongon-dong, Chongno-gu, Seoul 110-799, Korea

E-mail : kbumjoon@snu.ac.kr

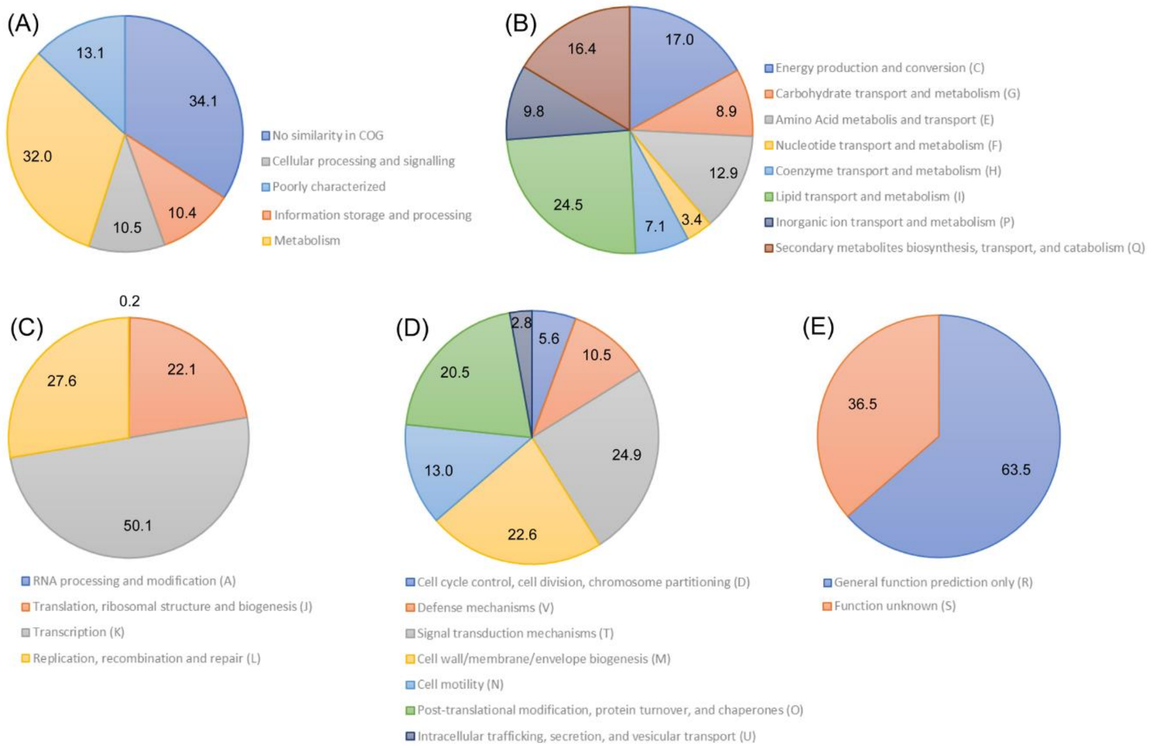

Supplementary Figure S1. Functional classification of the Mpg proteins based upon the COG database. (A) Representation of the general classification of the Mpg proteins in the COG database. Distribution of the Mpg proteins in the (B) ‘Metabolism’, (C) ‘Information storage and processing’ (ISP), (D) ‘Cell processing and signaling’ (CPS), and (E) ‘Poorly characterized’ categories.

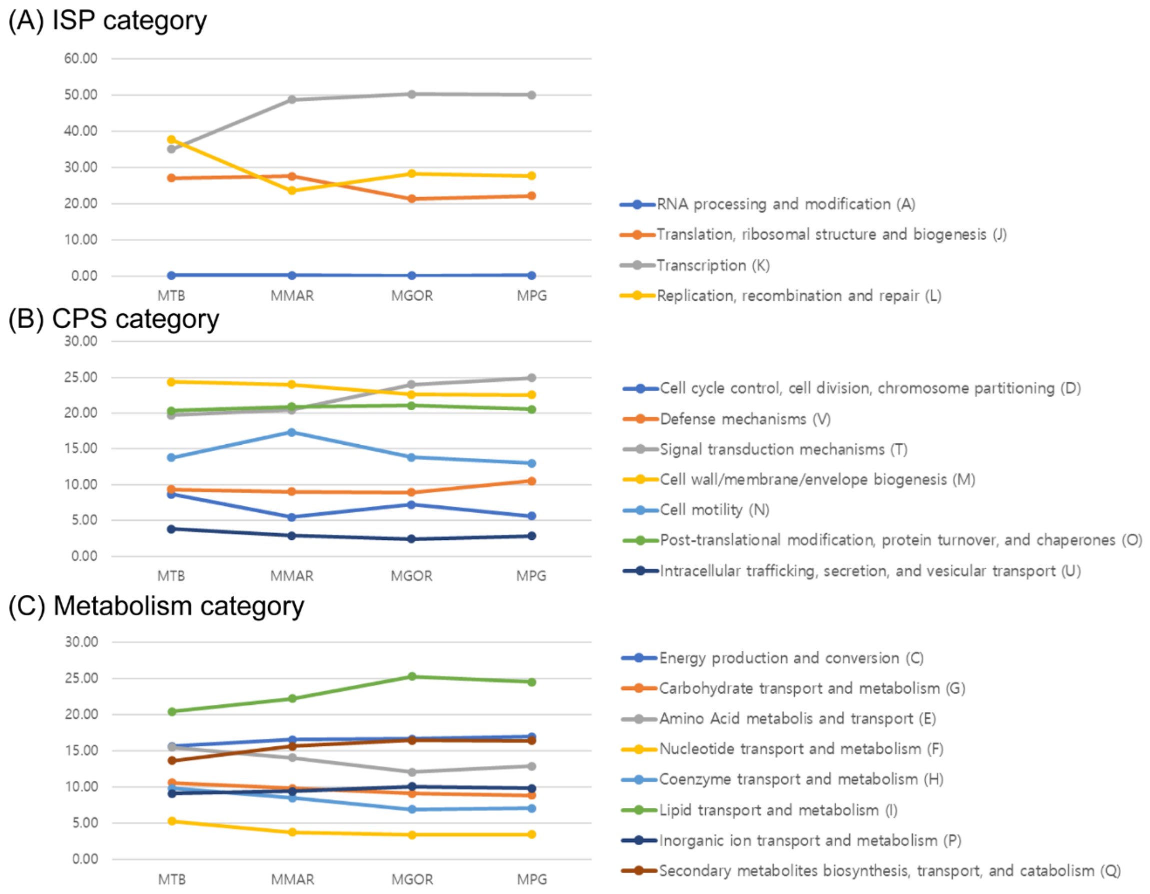

Supplementary Figure S2. Comparative analysis of the distribution of COG-based categorized proteins from the Mpg, *M. gordonae* DSM 44160^{T}, *M. marinum* M, and *M. tuberculosis* H37Rv strains. Comparison of the distribution of the genes involved in the category of (A) 'Information storage and processing' (ISP), (B) 'Cell processing and signaling' (CPS), and (C) 'Metabolism.' The X and Y axis represent the mycobacterial species and the proportion of genes involved in each subcategory (%), respectively.

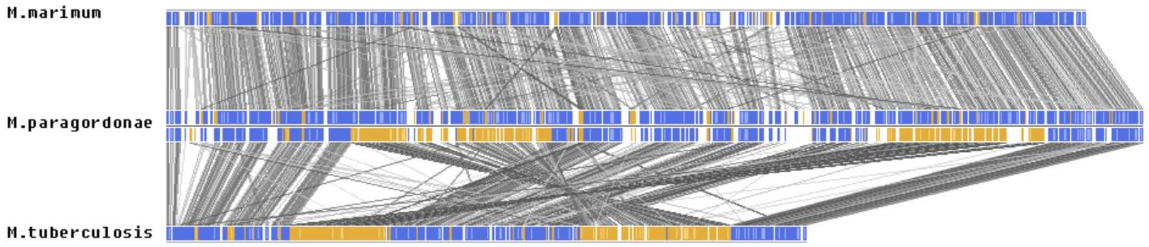

Supplementary Figure S3. Pairwise linear genomic comparison of Mpg, *M. marinum* M, and *M. tuberculosis* H37Rv. Orthologous genes are connected by lines and genes without an orthologous relationship are treated as gaps. Blue and yellow blocks indicate orthologs with the same and reversed order, respectively.

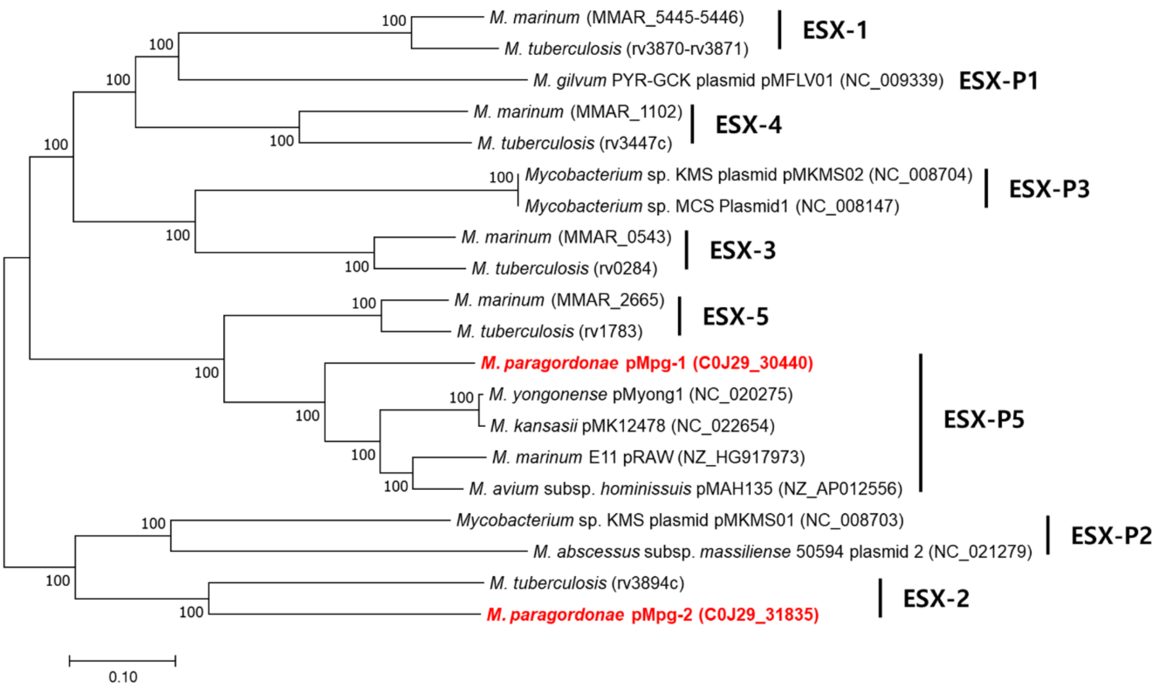

Supplementary Figure S4. Phylogenetic tree based on the EccC protein sequences of the pMpg-1 and -2 plasmids and the various EccC proteins from mycobacterial chromosomes (ESX-1 through -5 of *M. marinum* and *M. tuberculosis*) and plasmids (ESX-P1, pMFLV01 of *M. gilvum* PYR-GCK; ESX-P2, pMKMS01 of *Mycobacterium* sp. KMS and plasmid 2 of *M. abscessus* subsp. *massiliense* 50594; ESX-P3, pMKMS02 of *Mycobacterium* sp. KMS and plasmid 1 of *Mycobacterium* sp. MCS; ESX-P5, pMyong1 of *M. yongonense*, pMK12478 of *M. kansasii*, pRAW of *M. marinum* and pMAH135 of *M. avium* subsp. *hominissuis*).

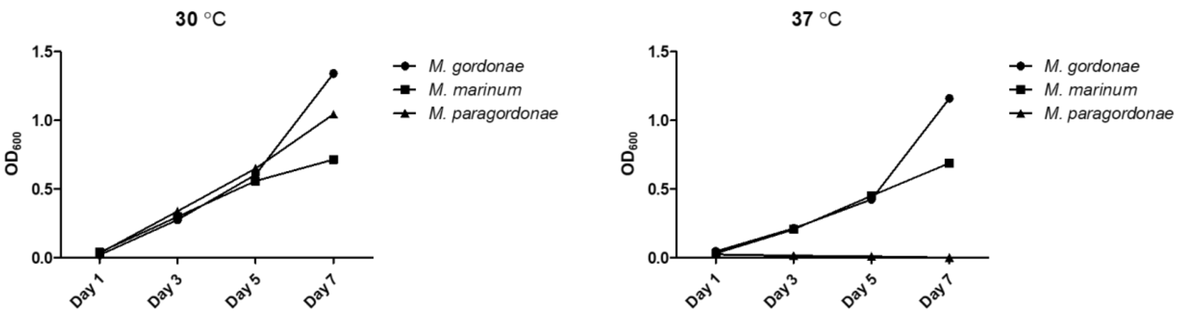

Supplementary Figure S5. The growth curve of three mycobacterial strains, Mpg, *M. gordonae* and *M. marinum* in 7H9 broth media supplemented with ADC for 7 days at 30 and 37 °C. To establish the growth curve, culture aliquots were taken at each time point and the OD_{600} was measured.

Supplementary Table S1. Comparison of average nucleotide identity (ANI) values among the Mpg and other mycobacterial strains.

ANI value (%)	Mpg*	Mgor	Mmar	Mtb
Mpg		86.68	78.12	78.58
Mgor			77.84	78.39
Mmar				78.86
Mtb				

*Mpg, *M. paragordonae* JCM 18565^T; Mgor, *M. gordonae* DSM 44160^T; Mmar, *M. marinum* M; Mtb, *M. tuberculosis* H37Rv^T.

Supplementary Table S2. BLAST analysis of ORFs in the novel ESX locus of pMpg-2 plasmid

Locus ID	Descriptions	Species	Similarities (%)
C0J29_31780	type VII secretion AAA-ATPase EccA	*Mycobacterium noviomagense*	55
C0J29_31785	type VII secretion protein EccE	*M. abscessus*	48
C0J29_31790	type VII secretion-associated serine protease	*M. abscessus*	61
C0J29_31795	type VII secretion integral membrane protein EccD	*M. abscessus*	52
C0J29_31800	MinD/ParA family protein	*M. abscessus*	60
C0J29_31805	ESX secretion-associated protein EspG	*M. abscessus*	53
C0J29_31810	Putative ESX protein	*Mycobacterium* sp.	38
C0J29_31815	Putative ESX protein	*M. avium*	63
C0J29_31820	PPE domain-containing protein	*M. abscessus*	52
C0J29_31825	PE2 protein	*M. abscessus*	63
C0J29_31830	Lsr2 family protein	*Nocardia otitidiscaviarum*	51
C0J29_31835	type VII secretion protein EccCb	*M. abscessus*	56
C0J29_31840	DNA translocase FtsK	*M. abscessus*	49
C0J29_31845	type VII secretion protein EccB	*M. abscessus*	59
